# Inferiorly Based Björk Flap and Accidental Decannulation in Surgical Tracheostomy: A Single-Institution Cohort Study and Meta-Analysis

**DOI:** 10.3390/medsci14040442

**Published:** 2026-07-27

**Authors:** Yeon Soo Kim, Sungryeal Kim, Jeon Yeob Jang, Yoo Seob Shin, Chul-Ho Kim

**Affiliations:** 1 Department of Otorhinolaryngology-Head & Neck Surgery, Korea University College of Medicine, Seoul 02841, Republic of Korea; 2 Department of Otorhinolaryngology-Head & Neck Surgery, Ajou University School of Medicine, Suwon 16499, Republic of Korea

**Keywords:** accidental decannulation, tracheostomy, inferior-based tracheal flap, safety protocol

## Abstract

**Objective**: Airway emergencies in patients with tracheostomies are life-threatening and may arise from tube obstruction, bleeding, or accidental decannulation owing to improper handling. We describe our institutional experience with measures to prevent accidental decannulation-related emergencies, including the inferior-based tracheal flap technique combined with peri-operative care bundles and its impact on reducing accidental decannulation-related morbidity and mortality. **Methods**: A retrospective review was conducted on 1485 patients who underwent open tracheostomy at Ajou University Hospital between January 2011 and February 2026. In 2015, a standardized institutional protocol, incorporating the inferior-based tracheal flap as the primary incision technique and a peri-operative care bundle, was introduced following a root-cause analysis. The cohort was divided into pre-protocol (2011–2014; *n* = 179) and post-protocol (2015–2026; *n* = 1306) periods, and a long-term-followable analytic subset (Björk flap *n* = 363; conventional vertical-incision *n* = 61) was used for per-outcome comparison. These data were integrated with prior comparative studies from the literature in a random-effects meta-analysis; six literature studies plus the institutional cohort were included in the qualitative synthesis, and those with extractable arm-specific event data contributed to the quantitative synthesis. Risk of bias was assessed with ROBINS-I and the Newcastle–Ottawa Scale, and the certainty of evidence was rated using GRADE. Pre-specified sensitivity analyses, including the Hartung–Knapp–Sidik–Jonkman adjustment, were performed. All analyses were performed in R using the metafor package. **Results**: Pre-protocol, accidental decannulation-related emergencies occurred one to two times per year, including at least one fatal event; post-protocol, only a single event occurred over 12 years, with no associated mortality. In the analytic subset, accidental decannulation occurred in 1/363 Björk patients (0.28%) versus 3/61 conventional patients (4.92%; odds ratio [OR] 0.05, 95% confidence interval [CI] 0.01–0.52). All six pooled meta-analytic domains were directionally favorable to the Björk flap. The primary outcome (accidental decannulation/tube displacement) did not reach statistical significance in the main analysis (pooled OR 0.44, 95% CI 0.03–5.86, *p* = 0.531, I^2^ = 77%, k = 3). A pre-specified sensitivity analysis excluding İnan 2025 (the heterogeneity-driving outlier) yielded an OR of 0.12 (95% CI 0.02–0.70, *p* = 0.019, I^2^ = 9%, k = 2); this result is exploratory and hypothesis-generating. Secondary outcomes, including stomal infection (OR 0.26), granulation (OR 0.15), tube blockage (OR 0.21), subcutaneous emphysema (OR 0.38), and bleeding (OR 0.59), all directionally favored the Björk flap. No tracheal stenosis or decannulation failure occurred in either arm of our cohort. Under GRADE, the certainty of evidence was very low for the primary outcome and low for the secondary outcomes. **Conclusions**: A standardized institutional safety bundle centered on the inferior-based tracheal flap was associated with a low rate of accidental decannulation-related emergencies and no related mortality over a 12-year post-implementation period; because the flap was introduced together with concurrent peri-operative and nursing changes, its independent contribution cannot be isolated. Meta-analytic synthesis showed directionally favorable effects across all complication domains, although the primary outcome reached significance only in an exploratory sensitivity analysis and the overall certainty of evidence was low to very low. These findings are hypothesis-generating and support the inferior-based tracheal flap as a reasonable option within a structured safety bundle for elective surgical tracheostomy, particularly in patients at higher risk for accidental decannulation; prospective comparative studies are warranted.

## 1. Introduction

Tracheostomy is one of the most commonly performed surgical procedures in head and neck surgery and in critical care medicine, with indications ranging from acute upper airway obstruction to long-term ventilatory support [1,2]. Although generally considered a safe procedure, tracheostomy carries a well-documented risk of serious complications. Among these, accidental decannulation, the unintentional displacement or dislodgment of the tracheostomy tube, represents one of the most dangerous events, particularly in the early postoperative period before a stable tracheocutaneous tract has formed [3].

In a large retrospective series of 1130 tracheostomy patients, tube decannulation or obstruction was responsible for four deaths, underscoring the potentially catastrophic nature of this complication [4,5,6]. Accidental decannulation in the immediate postoperative period poses an especially high risk because the tracheostomy tract has not yet epithelialized, and blind attempts at tube reinsertion can create false tracts into the paratracheal soft tissue, leading to fatal airway loss [7]. Device-related etiologies of tracheostomy emergencies include tube obstruction (mucous plugging, blood clots, collapsed or kinked tube) and tube displacement (accidental decannulation, partial dislodgement), and recognizing these early is critical to patient survival.

Multiple strategies have been proposed to mitigate the risk of accidental decannulation. From a postoperative care standpoint, these include proper tube securement with tracheostomy ties, implementation of nursing care bundles, risk stratification tools, and strict monitoring protocols [8,9,10,11,12,13]. Furthermore, standardized emergency algorithms, including the National Tracheostomy Safety Project algorithm, have been validated to guide first responders during decannulation emergencies [5].

From a surgical perspective, techniques such as placement of lateral stay sutures [14], skin-to-mucosa suturing, starplasty [15], and the Björk flap [16] have been described to create a more stable and accessible stoma. The Björk flap involves creating an inferiorly based tracheal cartilage flap sutured to the overlying skin, forming a mature stoma from the time of surgery [17,18,19,20]. Clinical evidence suggests that the Björk flap is associated with lower rates of post-tracheostomy complications compared with conventional incision methods, and comparative analyses have demonstrated reductions in intra-operative and early postoperative complications [21].

Despite these advantages, the Björk flap has not been universally adopted, in part owing to concerns regarding long-term stomal complications and variability in surgical technique. In our institution, accidental decannulation-related emergencies occurred approximately once every one to two years until the introduction of a structured institutional protocol in 2015. This protocol centered on the inferior-based tracheal cartilage flap technique, performed in the operating room under general anesthesia, combined with strict peri-operative management guidelines. In the present study, we report our 15-year institutional experience with this protocol and its impact on the reduction in accidental decannulation-related emergencies and mortality. A systematic review and random-effects meta-analysis of the literature was also conducted to evaluate the role of the inferior-based tracheal flap technique across multiple complication domains.

## 2. Materials and Methods

### 2.1. Protocol and Registration

This systematic review and meta-analysis was conducted in accordance with the Preferred Reporting Items for Systematic Reviews and Meta-Analyses (PRISMA) 2020 guidelines [22].

### 2.2. Search Strategy

A comprehensive electronic search was performed across three databases: PubMed/MEDLINE, EMBASE, and the Cochrane Central Register of Controlled Trials (CENTRAL) from database inception through December 31, 2025. The search strategy combined Medical Subject Headings (MeSH) terms and free-text keywords related to procedures, including “tracheostomy,” “tracheotomy”; Björk flap technique, including “Björk flap,” “Bjork flap,” “inferior-based flap,” “tracheal flap,” “tracheal cartilage flap”; and relevant outcomes, including “accidental decannulation,” “tube displacement,” “tracheal stenosis,” “complications.” No language restrictions were applied. Reference lists of included studies and relevant reviews were hand-searched. Complete database-specific search strings are provided in Appendix A. Risk of bias for included comparative studies was assessed independently by two reviewers using the ROBINS-I tool [23] and the Newcastle–Ottawa Scale, and the certainty of evidence for each pooled outcome was rated using the GRADE approach [24]. Because fewer than ten studies contributed to each outcome, formal small-study/publication-bias testing (funnel plot, Egger’s test) was not performed, in accordance with Cochrane guidance; publication bias therefore cannot be excluded.

### 2.3. Eligibility Criteria

Studies were included if they (1) enrolled adult patients (age ≥ 18 years) undergoing open surgical tracheostomy; (2) compared the inferior-based or Björk flap technique with any alternative conventional technique (vertical incision, horizontal H-incision, or tracheal window/excision); and (3) reported at least one of the pre-specified outcomes. Excluded were studies restricted to percutaneous dilational tracheostomy, pediatric populations (age < 18 years exclusively), case reports (n < 10), and studies without a comparator group (for the meta-analysis only; single-arm studies were retained for qualitative synthesis). When multiple publications arose from the same cohort, only the most recent or comprehensive report was included.

### 2.4. Outcomes

The primary outcome was the rate of accidental/unplanned decannulation (or tube displacement/false passage as the closest reported equivalent). Secondary outcomes included stomal infection, subcutaneous emphysema/pneumothorax, peri-operative bleeding, stomal granulation, tracheal stenosis, and tube blockage/mucus plug.

### 2.5. Study Selection and Data Extraction

Two independent reviewers (Y.S.K. and Y.S.S) screened titles and abstracts, followed by full-text review of eligible studies. Discrepancies were resolved by consensus or adjudication by a third reviewer (C.H.K.). Data were extracted using a standardized template capturing: study design, year, country, sample size, tracheostomy indication, surgical technique details, follow-up duration, and outcome data.

### 2.6. Statistical Analysis

To preserve the independence assumption of the random-effects model, only one outcome per study was retained within each outcome domain; when a single study reported multiple correlated endpoints within a domain (e.g., İnan 2025 reported “tube displacement” and “false passage” for the primary outcome domain; Chauhan 2023 reported “intra-operative” and “primary postoperative” bleeding), the more clinically representative endpoint of the domain was selected a priori. For studies with zero events in one arm, a Haldane–Anscombe continuity correction of 0.5 was applied [25]. Statistical heterogeneity was quantified using the Cochran Q statistic and I^2^ statistic; I^2^ > 50% was considered to represent substantial heterogeneity. All analyses used random-effects models (DerSimonian–Laird method), which are more conservative when heterogeneity is present. As a pre-specified sensitivity analysis, pooled estimates were additionally computed using the Hartung–Knapp–Sidik–Jonkman adjustment to account for uncertainty in the between-study variance in the setting of few, sparse-event studies [26]. Pre-specified sensitivity analyses excluded individual studies identified as heterogeneity drivers. Outcomes with zero events in both arms across all contributing studies were reported descriptively but not formally pooled. All analyses were performed using R version 4.3.2 (R Foundation for Statistical Computing, Vienna, Austria) using the metafor package and Python 3.11 (custom scripts for cross-validation).

### 2.7. Institutional Cohort: Design and Patient Population

In parallel with the systematic review, we conducted a retrospective, single-institution cohort study of 1485 consecutive patients who underwent open surgical tracheostomy at Ajou University Hospital between January 2011 and February 2026. Percutaneous dilational tracheostomies were excluded. Patients were stratified into pre-protocol (2011–2014; *n* = 179) and post-protocol (2015–2026; *n* = 1306) cohorts, corresponding to the institution of the inferior-based tracheal flap protocol and peri-operative management guidelines. A long-term-followable analytic subset comprising patients who underwent temporary tracheostomy with elective decannulation after resolution of the underlying condition (head and neck cancer ablative surgery, deep neck infection, acute epiglottitis, or trauma) was identified for per-outcome comparison: Björk flap group *n* = 363; conventional vertical-incision group *n* = 61. Patients in whom long-term follow-up was not possible (e.g., long-term tracheostomy dependence, mortality from primary disease, or loss to follow-up) were excluded from the per-outcome analysis. The institutional cohort was included as a data point in the meta-analysis.

### 2.8. Institutional Protocol

**Figure 1:**
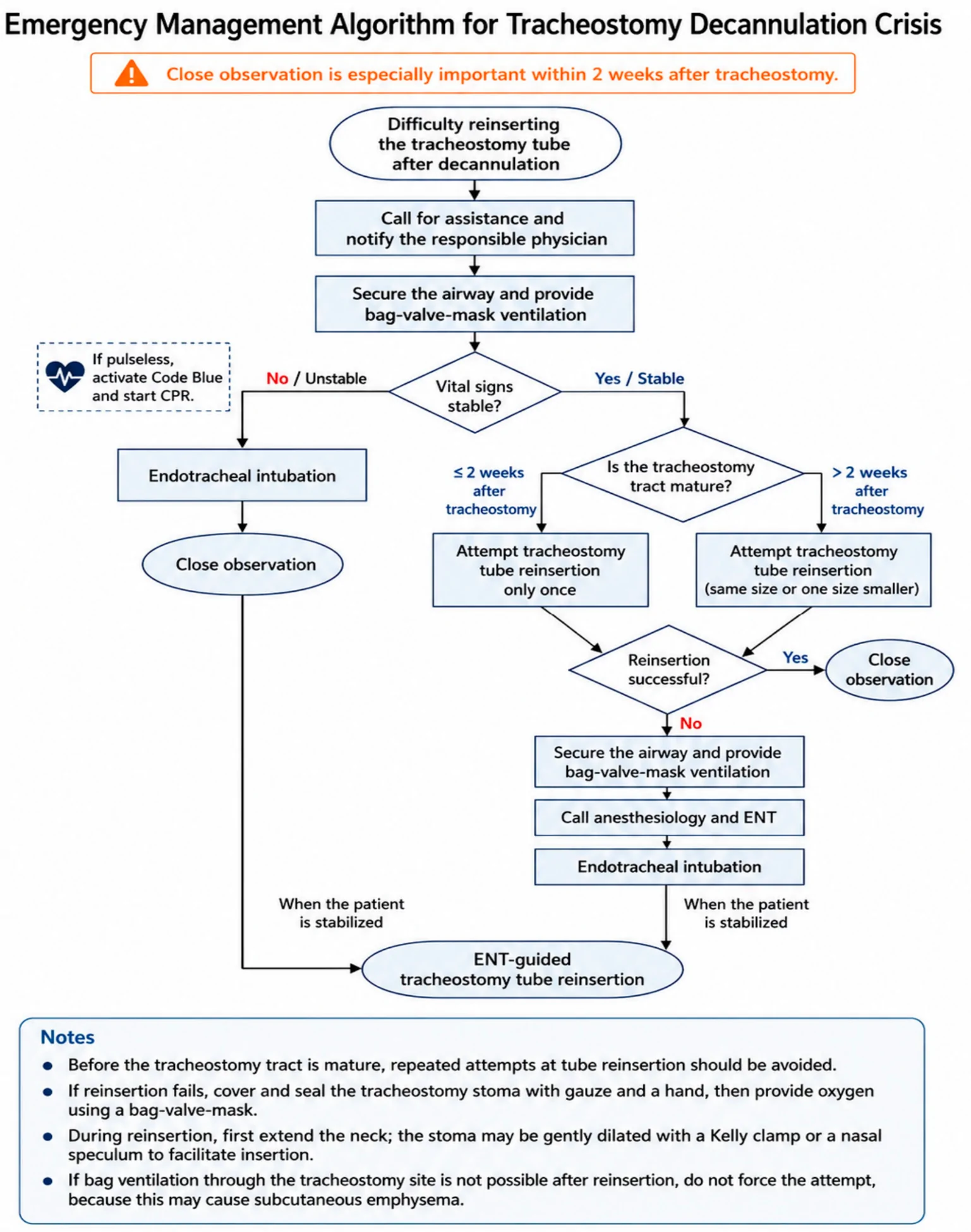
Institutional emergency-management algorithm for tracheostomy accidental decannulation, developed by the authors for nursing and medical staff education following protocol implementation in 2015. The algorithm was informed conceptually by established tracheostomy-safety principles, including those of the National Tracheostomy Safety Project (NTSP) [27]; no copyrighted material was reproduced.

In 2015, our institution introduced a comprehensive tracheostomy safety protocol following a root cause analysis (RCA) conducted after a fatal accidental decannulation event. The protocol included four key components: (1) performance of all elective and semi-elective tracheostomies in the operating room under general or local anesthesia; (2) adoption of the inferior-based tracheal cartilage flap as the standard incision technique; (3) avoidance of tracheostomy tube change in the immediate postoperative period; and (4) secure fixation of the tube with a tracheostomy tie, with the flap sutured to the skin. Nursing and medical staff were educated on an institutional accidental decannulation response algorithm (Figure 1) developed by the authors and informed conceptually by established tracheostomy-safety principles, including those of the National Tracheostomy Safety Project [27].

### 2.9. Surgical Technique: Inferior-Based Tracheal Flap

**Figure 2:**
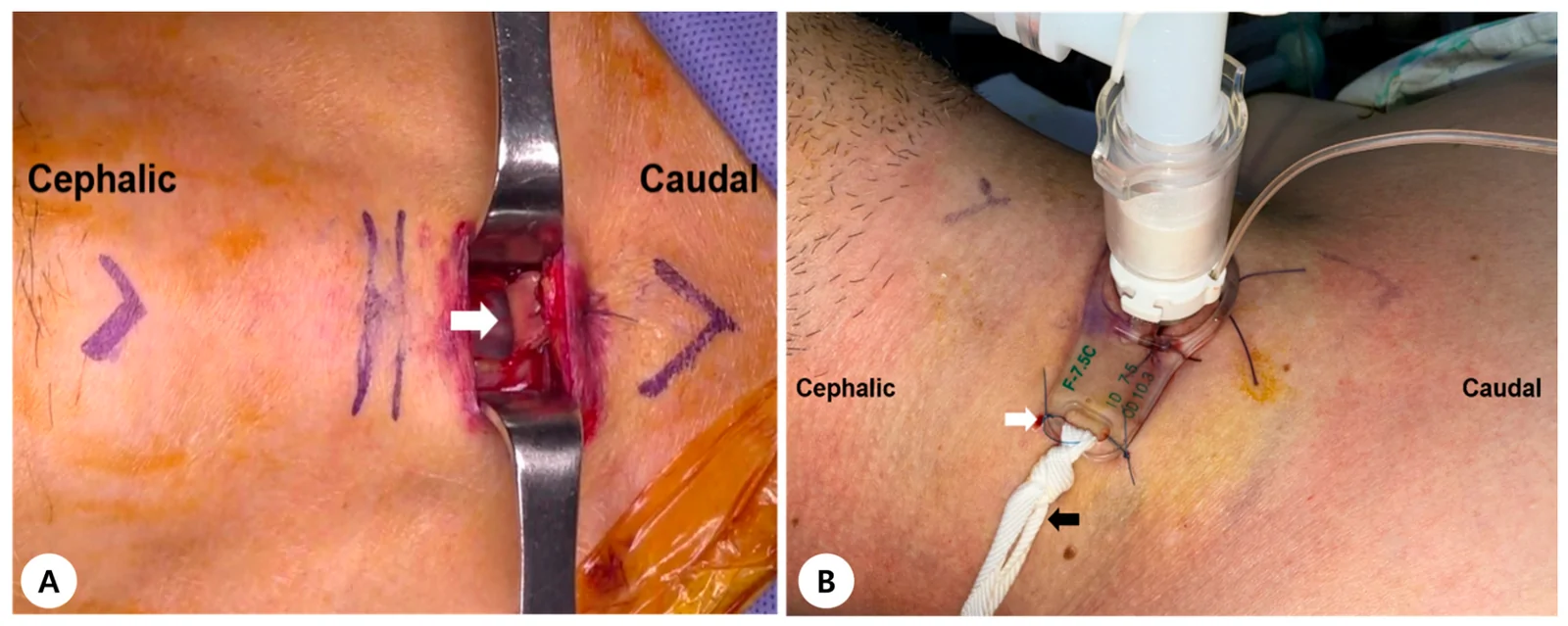
(**A**) Intra-operative photograph of the inferior-based tracheal cartilage flap technique. An inferiorly based, inverted U-shaped cartilaginous flap is created by a horizontal incision at the inferior border of the second and/or third tracheal ring with two short lateral vertical limbs, leaving the inferior margin intact as the pedicle. The flap is reflected inferiorly and secured with a single flap-to-skin maturation suture using 3-0 or 4-0 absorbable braided multifilament suture, creating a stable inferior shelf that serves as an anatomic guide for tube reinsertion. (**B**) Tube securement strategy: a circumferential tracheostomy tie with a skin suture through the tube flange; optional lateral stay sutures (2-0 silk) through the tracheal wall may be added at the surgeon’s discretion but are not required.

After standard exposure of the anterior trachea and division of the thyroid isthmus when necessary, the inferior-based tracheal flap was created as follows. Using a No. 15 blade, a horizontal incision was made through the anterior tracheal wall at the inferior border of the second or third tracheal ring and continued as two short vertical limbs along the lateral margins, leaving the inferior margin intact as the pedicle to create an inferiorly based, inverted U-shaped cartilaginous flap. The flap was carefully elevated and reflected inferiorly, and its inferior free edge was sutured to the inferior skin margin using 3-0 or 4-0 absorbable braided multifilament sutures (Vicryl^®^ (Ethicon Inc., Somerville, NJ, USA), Figure 2A). This maneuver creates a stable, epithelialized inferior ledge that serves as a guide for tube reinsertion and prevents the tube from being directed into a false paratracheal tract in the event of accidental decannulation. A single flap-to-skin maturation suture is sufficient to establish this guided tract, and multiple fixation sutures are not obligatory. Optionally, lateral stay sutures (2-0 silk) may be placed through the tracheal wall on either side of the stoma and secured externally to the skin as an additional safeguard, at the surgeon’s discretion; in our practice these were not routinely required. The tracheostomy tube was further secured with a circumferential tracheostomy tie and a skin suture through the flange (Figure 2B).

### 2.10. Institutional Cohort Outcome Measures

The primary outcome was the incidence of accidental decannulation-related emergencies, defined as any unintentional displacement of the tracheostomy tube requiring emergent intervention, with particular attention to early events occurring within the first 14 postoperative days, the period of greatest risk before tract maturation. Secondary outcomes included decannulation-related mortality, incidence of false tract formation, and long-term complications, including tracheal stenosis, stomal granulation, and tracheocutaneous fistula. For the per-outcome analytic subset, only stomal granulation and secondary hemorrhage cases requiring surgical intervention were counted. Patients were followed for a minimum of six months post-decannulation or until last clinical contact.

## 3. Results

### 3.1. Institutional Cohort Results

**Figure 3:**
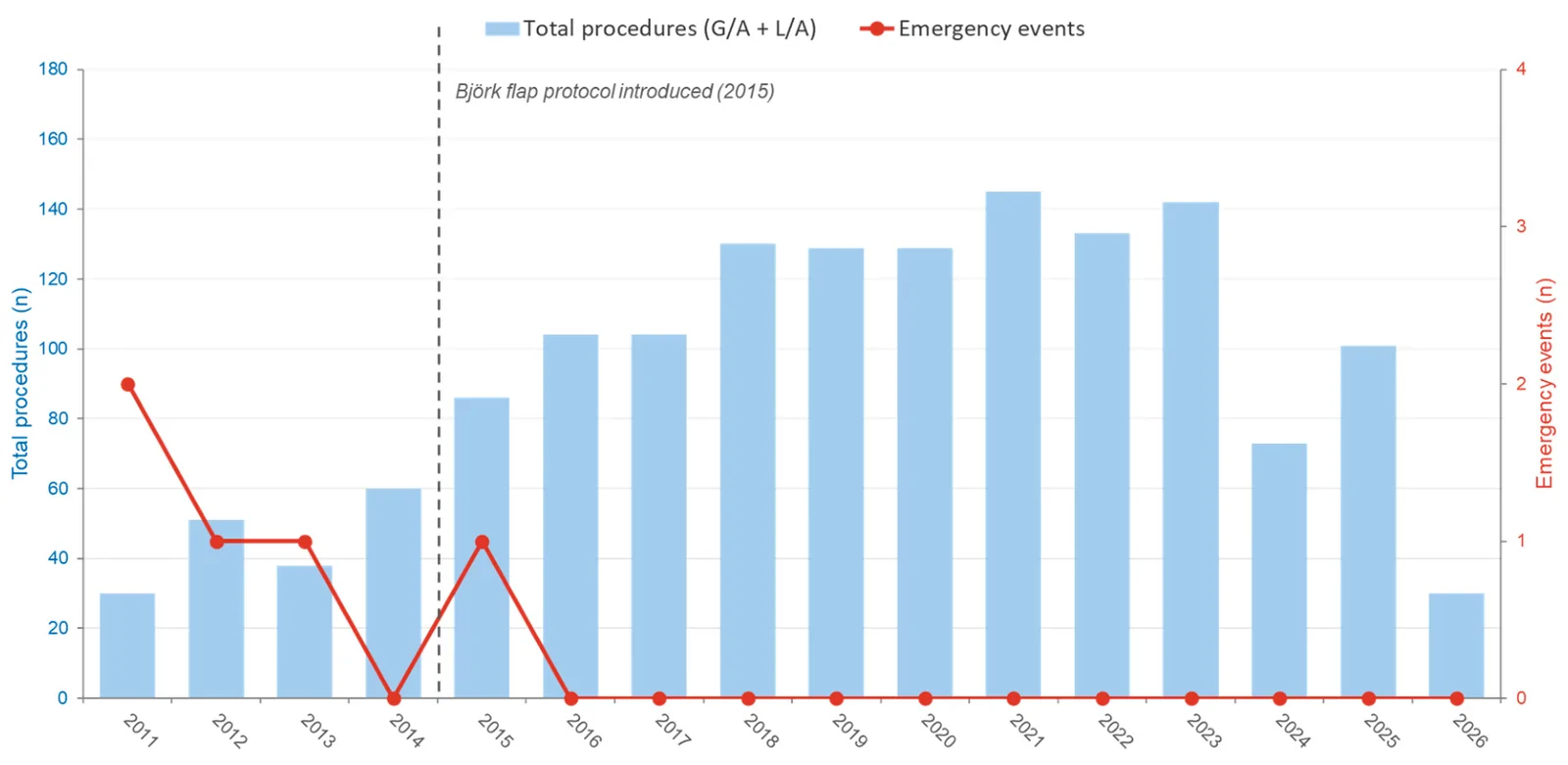
Timeline of accidental decannulation-related emergencies at our institution before and after protocol implementation in 2015. Pre-protocol period (2011–2014): one to two events per year, including at least one fatal event. Post-protocol period (2015–2026): one non-fatal event over 12 years (0.08 per 100 procedures).

Prior to protocol implementation (2011–2014; *n* = 179), accidental decannulation-related emergencies occurred at a frequency of approximately one to two events annually, including at least one fatal event. Following implementation of the inferior-based tracheal flap protocol in 2015 (post-protocol cohort: *n* = 1306, follow-up through 2026), accidental decannulation events were reduced to a single event over the 12-year period, with no associated mortality (Figure 3). Expressed per 100 procedures, the early accidental-decannulation rate fell from 2.23 per 100 (4/179) in the pre-protocol period to 0.08 per 100 (1/1306) in the post-protocol period; within the analytic subset, the incidence-rate ratio was 0.04 (95% CI 0.005–0.376), Fisher exact *p* = 0.002. In all post-protocol cases in which accidental decannulation occurred, the tube was successfully replaced without the creation of a false tract, aided by the structurally stable inferior shelf within the stoma created by the flap.

For the per-outcome analytic subset (Björk flap *n* = 363; conventional vertical-incision *n* = 61), complication rates are summarized in Table 1. Patients excluded from the analytic subset were those for whom long-term follow-up was not possible or those requiring chronic tracheostomy dependence (precluding evaluation of decannulation-related outcomes); the analytic subset comprised patients with temporary tracheostomy for head and neck cancer ablative surgery, deep neck infection, acute epiglottitis, or trauma, followed by elective decannulation after resolution of the underlying condition. For stomal granulation and secondary hemorrhage, only cases requiring surgical intervention were counted. No case of persistent tracheocutaneous fistula occurred in either group (0/363 Björk vs. 0/61 conventional). No statistically significant increase in long-term complications (tracheal stenosis, stomal granulation, secondary hemorrhage, tracheocutaneous fistula, or decannulation failure) was observed in the post-protocol Björk flap group.

### 3.2. Systematic Review: Study Selection

**Figure 4:**
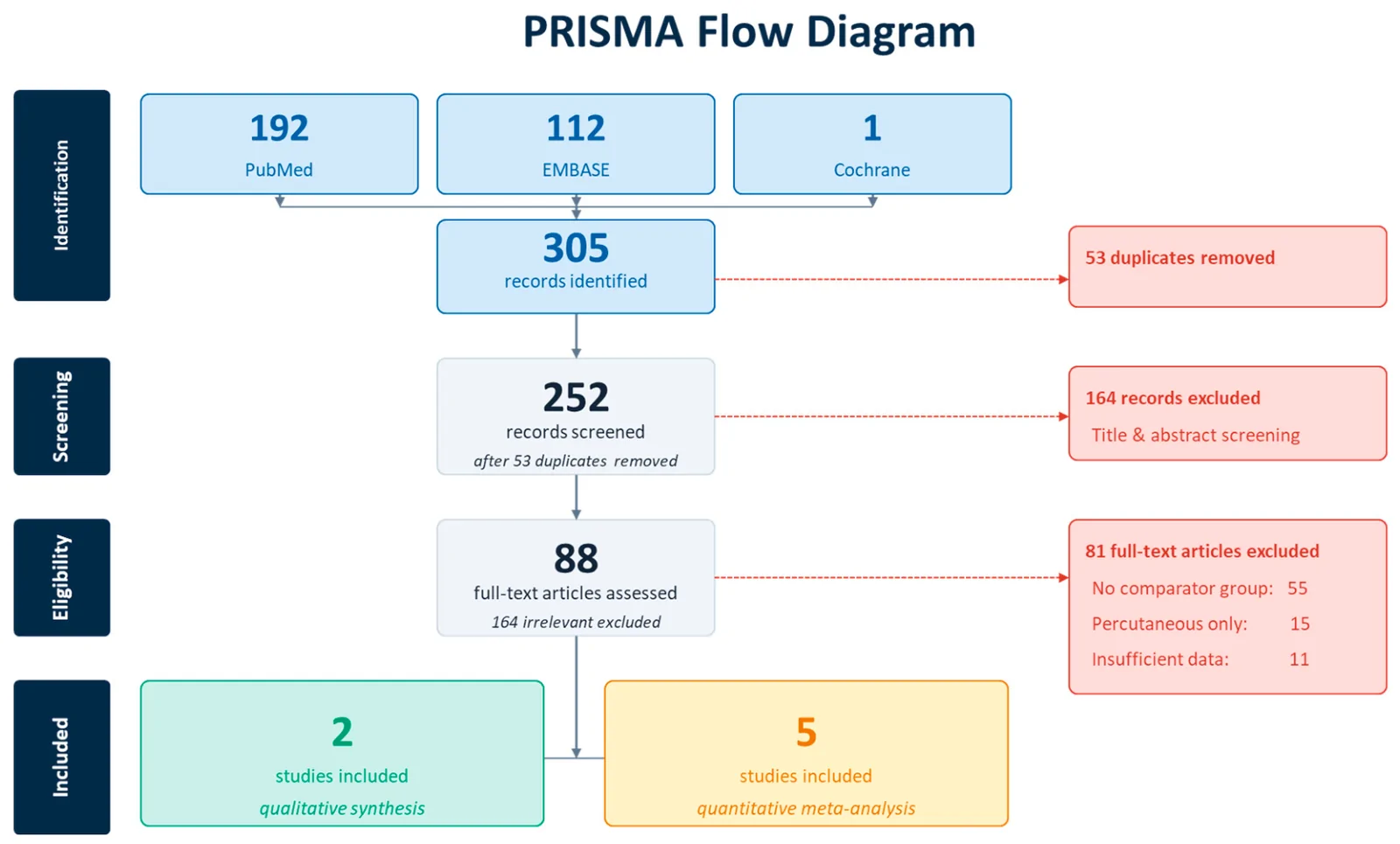
PRISMA 2020 flow diagram for systematic review and meta-analysis. From 305 records identified across three databases, seven studies were retained for qualitative synthesis and five for quantitative meta-analysis (four prior comparative studies plus our institutional cohort).

The initial electronic search identified 305 records across three databases (PubMed: 192; EMBASE: 112; Cochrane: 1). After removal of 53 duplicates, 252 records underwent title and abstract screening. Following exclusion of 164 irrelevant records, 88 full-text articles were assessed for eligibility; of these, 81 were excluded (no comparator group: 55; percutaneous technique only: 15; insufficient data: 11). A total of six comparative studies from the literature were retained, plus our institutional cohort, yielding seven datasets for qualitative synthesis. Of these, five datasets (Chauhan 2023 [28], Kennedy 2021 [29], Silva-Nash 2023 [30], İnan 2025 [31], and our institutional cohort) had arm-specific event counts extractable for quantitative meta-analysis; two additional studies (Çelikal 2022 [32], pediatric population, included for qualitative completeness only; Marini 2023 [33], decannulation counts not accessible from available data) were retained in qualitative synthesis. The PRISMA flow diagram is presented in Figure 4. Risk-of-bias assessments (ROBINS-I, Newcastle–Ottawa Scale) and the GRADE certainty ratings are summarized in the Supplementary Materials. Characteristics of the included candidate studies are summarized in Table 2.

### 3.3. Meta-Analysis: Study Population and Pooled Dataset

The quantitative meta-analysis comprised five comparative datasets from Chauhan et al. [28], Kennedy et al. [29], Silva-Nash et al. [30], İnan et al. [31], and our institutional cohort, yielding a total of 870 Björk flap and 487 conventional tracheostomy cases across six analyzable outcome domains. Our cohort contributed the largest single dataset (363 Björk vs. 61 conventional) and provided novel data concerning accidental decannulation, an outcome not separately reported in most prior series.

### 3.4. Primary Outcome: Accidental Decannulation/Tube Displacement

In our institutional cohort, accidental decannulation occurred in 1 of 363 Björk patients (0.28%) versus 3 of 61 conventional patients (4.92%), corresponding to an OR of 0.05 (95% CI 0.01–0.52), a 95% relative reduction. When pooled with two additional comparative datasets (Silva-Nash 2023, false passage; İnan 2025, tube displacement), the random-effects estimate was an OR of 0.44 (95% CI 0.03–5.86, *p* = 0.531, k = 3) with substantial heterogeneity (I^2^ = 77%). This primary pooled estimate did not reach statistical significance, and we regard it as the principal result for this outcome. Under the Hartung–Knapp–Sidik–Jonkman adjustment, the confidence interval widened further, reinforcing the uncertainty of this sparse-event estimate. The wide confidence interval reflects the divergent direction of effect contributed by İnan 2025 (tube displacement OR 3.92, 95% CI 0.63–24.26), which was the dominant heterogeneity driver. A pre-specified sensitivity analysis excluding İnan 2025 yielded an OR of 0.12 (95% CI 0.02–0.70, *p* = 0.019, k = 2) with reduced heterogeneity (I^2^ = 9%); we present this strictly as an exploratory, hypothesis-generating analysis rather than as confirmatory evidence of efficacy, noting that İnan 2025 uniquely defined the event as post-reinsertion tube displacement rather than early accidental decannulation or false passage.

### 3.5. Secondary Outcome

#### 3.5.1. Bleeding/Hemorrhage

Four datasets contributed bleeding outcomes. The pooled estimate favored the Björk flap (OR 0.59, 95% CI 0.18–1.88, *p* = 0.370, I^2^ = 46%) but did not reach statistical significance. Chauhan 2023 (primary postoperative hemorrhage 0/30 vs. 7/30, OR 0.05) and our cohort (OR 0.33) showed strong protective effects, while Silva-Nash 2023 was intermediate (OR 0.63), and Kennedy 2021 reported a numerically unfavorable signal (postoperative bleeding OR 1.49, 95% CI 0.56–4.01). The Kennedy 2021 result likely reflects confounding by indication, as the authors explicitly noted that Björk flaps were preferentially applied to patients with elevated body mass index (BMI) and prolonged ventilation needs at their institution. In our cohort, secondary hemorrhage occurred in 2 of 363 Björk patients (0.55%) versus 1 of 61 conventional patients (1.64%).

#### 3.5.2. Stomal Infection/Surgical Site Infection

The pooled estimate across three datasets was an OR of 0.26 (95% CI 0.03–1.98, *p* = 0.192) with high heterogeneity (I^2^ = 77%). Chauhan 2023 reported an exceptionally strong protective effect (3/30 vs. 22/30, OR 0.04, 95% CI 0.01–0.17), which is the largest individual effect size observed in the entire meta-analysis. Silva-Nash 2023 showed a directionally consistent but weaker signal (OR 0.35, 95% CI 0.10–1.23), while İnan 2025 reported a numerically opposite point estimate (OR 2.52, 95% CI 0.15–41.17) based on very few events (1/47 vs. 1/117). When Chauhan 2023 was excluded as a potential outlier, the pooled estimate attenuated to an OR of 0.62 (95% CI 0.11–3.57, *p* = 0.596), indicating that the apparent benefit in the primary analysis was substantially driven by a single high-quality, but small, prospective series.

#### 3.5.3. Other Secondary Outcomes

Across the remaining three outcome domains, all pooled estimates favored the Björk flap directionally without reaching statistical significance. Subcutaneous emphysema/pneumothorax (k = 2): pooled OR 0.38 (95% CI 0.07–2.10, *p* = 0.268, I^2^ = 49%). Granulation/suprastomal pathology (k = 2): pooled OR 0.15 (95% CI 0.01–1.47, *p* = 0.103, I^2^ = 78%). Tube blockage/mucus plug (k = 2): pooled OR 0.21 (95% CI 0.01–5.22, *p* = 0.343, I^2^ = 81%). In each case, Chauhan 2023 contributed a strong protective signal that was incompletely replicated in other series, accounting for the high heterogeneity. In our cohort, stomal granulation occurred in 6 of 363 Björk cases (1.65%) versus 2 of 61 conventional cases (3.28%), corresponding to an OR of 0.50 (95% CI 0.10–2.51).

#### 3.5.4. Zero-Event Outcomes: Tracheal Stenosis, Decannulation Failure, and Tracheocutaneous Fistula

In our cohort, neither tracheal stenosis nor decannulation failure occurred in either group (0/363 in the Björk arm and 0/61 in the conventional arm), precluding odds ratio estimation. Likewise, no persistent tracheocutaneous fistula was recorded in either group (0/363 vs. 0/61); this zero-zero distribution similarly precluded formal effect estimation but is reported descriptively. Although a formal pooled effect cannot be derived, the complete absence of these structural late complications across 363 consecutive Björk flap cases over the 15-year study period represents a clinically meaningful safety signal. This is particularly relevant given that historical concerns regarding the Björk flap have centered on theoretical risks of tracheal cartilage weakening and late stenosis from inferior-based flap creation, as well as persistent tracheocutaneous fistula from flap-to-skin maturation; our data argue against these concerns in a high-volume tertiary practice.

#### 3.5.5. Direction of Effect and Heterogeneity

**Figure 5:**
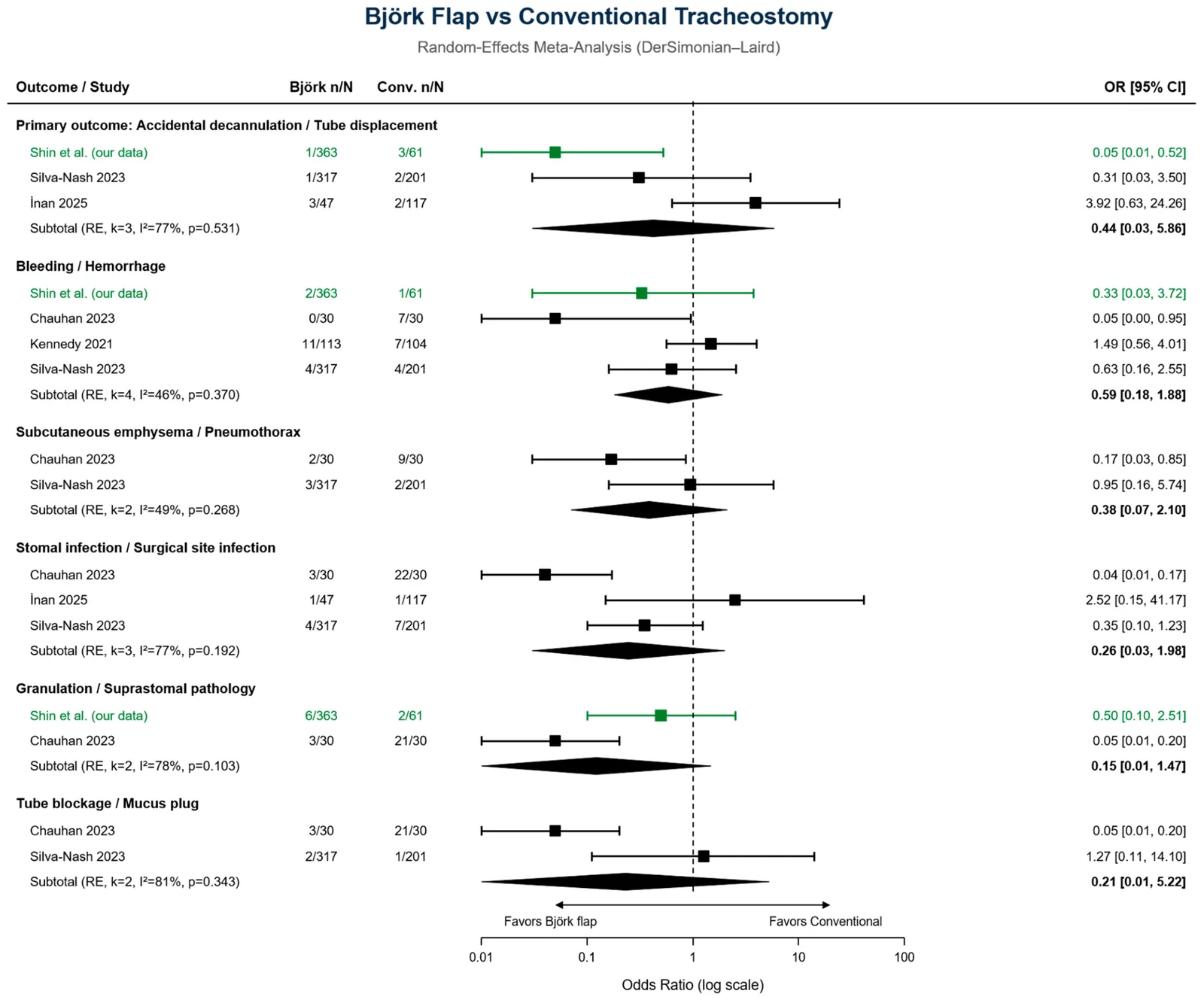
Forest plot of the random-effects (DerSimonian–Laird) meta-analysis comparing Björk flap versus conventional tracheostomy across six outcome domains. Domain headers indicate outcome category; individual rows show contributing studies (Shin et al., our data, in green; other studies in black). Squares represent point estimates of individual study–outcome pairs with horizontal lines indicating 95% confidence intervals; the size of each square is proportional to the inverse-variance weight. Diamonds represent the pooled random-effects estimate for each domain, with width corresponding to the 95% confidence interval. The vertical dashed line indicates the null effect (odds ratio = 1); odds ratios < 1 favor the Björk flap. Contributing studies are cited by author and year within the figure and correspond to the following references: Chauhan and Kumar, 2023 [28]; Kennedy et al., 2021 [29]; Silva-Nash et al., 2023 [30]; İnan et al., 2025 [31]. CI, confidence interval; OR, odds ratio; RE, random-effects.4. Discussion.

All six pooled domains showed directionally favorable estimates for the Björk flap, with pooled odds ratios ranging from 0.15 to 0.59 (Figure 5). None reached statistical significance in the main pooled analysis, attributable to substantial between-study heterogeneity (I^2^ range 46–81%). However, sensitivity analyses removing identified outlier studies, particularly İnan 2025 for the primary outcome, restored statistical significance with substantial reduction in heterogeneity (sensitivity-adjusted primary OR 0.12, 95% CI 0.02–0.70, *p* = 0.019, I^2^ = 9%, k = 2). This pattern suggests that the underlying biological effect is more robust than the main analysis indicates and that observed heterogeneity reflects methodological differences in outcome definition and case-mix rather than true biological variation.

## 4. Discussion

This study presents two complementary lines of evidence regarding the inferior-based Björk flap technique in surgical tracheostomy. First, a 15-year single-institution experience demonstrated that a comprehensive safety protocol centered on the Björk flap was associated with a marked reduction in accidental decannulation-related emergencies and mortality: in 1306 post-protocol tracheostomies over 12 years, only one accidental decannulation event occurred (0.08 per 100 procedures), compared with one to two events annually, including at least one fatal event during the 4-year pre-protocol period. Second, an updated random-effects meta-analysis integrating our institutional data with prior comparative studies from the literature showed that all six analyzable outcome domains were directionally favorable to the Björk flap (pooled OR range 0.15–0.59). The primary outcome of accidental decannulation/tube displacement did not reach statistical significance in the main analysis (OR 0.44, 95% CI 0.03–5.86, *p* = 0.531); significance emerged only in an exploratory sensitivity analysis following exclusion of a single heterogeneity-driving outlier study (OR 0.12, 95% CI 0.02–0.70, *p* = 0.019), and this result should be regarded as hypothesis-generating.

The mechanistic rationale for a protective effect against accidental decannulation is well established. Accidental decannulation accounts for approximately half of airway-related deaths in critical care settings, with a reported incidence of 4.2 events per 1000 tracheostomy-days [6,34,35]. By suturing an inferiorly based tracheal cartilage flap to the skin edge, a guided pre-formed tract is created from skin to trachea even before stoma maturation. Should accidental dislodgement occur, the tract remains patent and identifiable, allowing rapid reinsertion by nursing staff or non-surgical responders, thereby transforming a potentially life-threatening airway emergency into a controllable bedside event. The low accidental decannulation rate observed in our 363-case analytic subset (0.28%, 95% CI 0.01–1.53 per 100) and the absence of decannulation-related mortality across 1306 post-protocol patients are consistent with this mechanism, although the observational design precludes attribution of these outcomes to the flap alone.

A central methodological observation of the meta-analysis is the substantial between-study heterogeneity (I^2^ 46–81%) across nearly all outcome domains [26,36]. This heterogeneity is not random; it reflects identifiable methodological differences. Chauhan 2023 [28], a small prospective study (n = 30 per arm), contributed disproportionately strong protective effects for nearly every outcome, with odds ratios of 0.04–0.05 for stomal infection, granulation, primary hemorrhage, and tube blockage. However, these findings were incompletely replicated in larger series. Kennedy 2021 reported a numerically unfavorable bleeding signal (OR 1.49), which the original authors attributed to confounding by indication, as Björk flaps were selectively applied to higher-risk patients with elevated BMI and prolonged ventilation needs [34]. İnan 2025 [31] tube displacement (OR 3.92) was inconsistent with displacement outcomes reported in other series and with the strong protective effect observed in our cohort, suggesting either an outcome-definition difference, a true center-level effect, or chance variation in a small sample. Random-effects pooling appropriately widens confidence intervals to reflect this uncertainty, but the underlying directional concordance across all six domains is a robust signal that may be diluted by the conservative statistical model. To address this, we applied a one-outcome-per-study rule within each domain, preserving the independence assumption underlying the random-effects model.

Our findings extend the prior literature in important ways. Au and colleagues, in their 2017 narrative review [16], concluded that the evidence base was insufficient to recommend routine Björk flap use despite favorable safety signals. Our quantitative synthesis confirmed a consistent directional benefit across six domains; however, statistical significance for the primary outcome emerged only in an exploratory sensitivity analysis, and the overall certainty of evidence remained low to very low under GRADE. Additionally, it tempers earlier optimism by demonstrating that the magnitude of effect is highly study-dependent. The complete absence of late tracheal stenosis and decannulation failure across 363 consecutive Björk flap cases is nonetheless noteworthy [37,38,39,40]. Historical concerns regarding the Björk flap have centered on theoretical risks of flap dislodgement causing airway obstruction and progressive stenosis from cartilage weakening. Our data, representing one of the largest reported single-institution Björk flap series, argue against these long-term safety concerns in a high-volume tertiary practice. The juxtaposition of zero stenosis events in our Björk cohort against the 1.9% incidence reported in Goldenberg’s mixed-technique series supports the long-term safety of the technique when standardized.

Beyond the surgical technique itself, our institutional experience underscores that technique adoption alone may be insufficient without a comprehensive peri-operative care bundle [41,42,43]. Our protocol combined the Björk flap with operating-room performance under controlled anesthesia, avoidance of early tube change, secure fixation of the tube, and staff education on a standardized response algorithm. We emphasize that the flap-to-skin maturation itself provides the principal safeguard, and that a single flap-to-skin suture is sufficient; additional lateral fixation sutures are optional rather than obligatory. The dramatic pre/post-protocol difference, from one to two events annually to a single event over 12 years, coincided with concurrent secular changes over the 15-year period—enhanced ICU nurse staffing, structured tracheostomy nursing education, continuous capnographic monitoring, newer tube designs, and heightened institutional awareness after the index sentinel event—and therefore most likely reflects the synergistic effect of a multifaceted safety bundle rather than the surgical technique in isolation. This observation is consistent with broader patient-safety literature emphasizing systems-level interventions over isolated technical changes.

Certain limitations warrant explicit discussion. First, all studies included in the meta-analysis were retrospective and observational, with inherent risk of confounding by indication that meta-analytic pooling cannot fully address. Second, surgical technique standardization across centers was variable; Chauhan 2023 [28], with the largest effect sizes, was a small prospective study and may reflect genuine technique benefit and chance variation. Third, outcome definitions (“tube displacement” vs. “false passage” vs. “accidental decannulation”) differed across studies, requiring interpretive grouping into composite endpoints, which likely contributed to the observed heterogeneity. Fourth, the two zero-event safety outcomes (tracheal stenosis, decannulation failure) could not be formally pooled, representing an analytic limitation that simultaneously constitutes a reassuring safety signal. Fifth, the institutional pre/post-protocol comparison was subject to temporal confounding from concurrent improvements in nursing care, ICU practice, and monitoring technology over the 15-year study period. Sixth, no included study was a randomized controlled trial, and prospective randomized comparison with sufficient sample size for accidental decannulation as a primary endpoint remains the highest-priority evidence gap. Seventh, the reduction in the institutional cohort from 1485 screened patients to a 424-patient analytic subset introduces a risk of selection bias, and patient-level covariates—age, indication, ICU versus ward setting, prior neck irradiation, and prior head-and-neck cancer surgery—were incompletely captured and could not be fully adjusted for, so residual confounding cannot be excluded. Eighth, because fewer than ten studies contributed to each outcome, formal publication-bias testing was not feasible, and publication bias cannot be excluded. Finally, the review was not prospectively registered in PROSPERO or any other registry, which is acknowledged as a limitation.

Despite these limitations, the convergence of directional evidence supports the inferior-based Björk flap, applied within a structured safety bundle, as a reasonable option in appropriately selected patients rather than as an established standard. Our findings are most directly applicable to patients at higher risk for accidental decannulation, including those with obesity, difficult cervical anatomy, prolonged mechanical ventilation requirements, or care primarily by non-surgical staff. The technique adds minimal operative time and no specialized equipment, and—because a single flap-to-skin suture is sufficient—does not mandate additional fixation sutures, while consistently associating with directionally favorable outcomes across multiple complication categories. Future research should prioritize standardized outcome definitions, employ prospective registry data with sufficient sample size for individual-outcome analysis, and ideally perform randomized comparison with adequate power for low-incidence endpoints such as accidental decannulation.

## 5. Conclusions

A 15-year single-institution experience demonstrated that a comprehensive safety bundle centered on the inferior-based tracheal (Björk) flap was associated with a low rate of accidental decannulation-related emergencies and no related mortality, with event frequency declining from one to two annually (including at least one fatality) in the pre-protocol period to a single non-fatal event over 12 years in the post-protocol period; because the flap was introduced alongside concurrent peri-operative and nursing changes, its independent contribution cannot be isolated. In a per-outcome analytic subset, accidental decannulation occurred less frequently in the Björk group (0.28% vs. 4.92%; OR 0.05, 95% CI 0.01–0.52). An updated random-effects meta-analysis incorporating our cohort with prior comparative studies showed directionally favorable effects across all six analyzable complication domains; the primary outcome did not reach significance in the main analysis and became significant only in an exploratory sensitivity analysis (OR 0.12, 95% CI 0.02–0.70, *p* = 0.019), with low-to-very-low GRADE certainty overall. The complete absence of late tracheal stenosis and decannulation failure across our Björk flap experience supports the long-term safety of the technique. Within a structured peri-operative safety bundle, the inferior-based tracheal flap is a safe and technically reproducible option that may meaningfully reduce accidental decannulation-related airway emergencies. While the observational design and statistical heterogeneity call for measured interpretation, the consistent benefit across outcomes and the absence of decannulation-related mortality over a 15-year period support its adoption as a reasonable strategy in appropriately selected patients. Prospective studies are warranted to confirm these findings.

## Figures and Tables

**Table 1:** Complication rates in the Ajou institutional cohortd.

Outcome	Conventional (*n* = 61)	Björk (*n* = 363)
Accidental decannulation	3 (4.92%)	1 (0.28%)
Stomal granulation	2 (3.28%)	6 (1.65%)
Secondary hemorrhage	1 (1.64%)	2 (0.55%)
Tracheal stenosis	0	0
Decannulation failure	0	0
Tracheocutaneous fistula	0	0

**Table 2:** Characteristics of included candidate studies.

Study	Design/Population	Intervention (Björk)	Comparator	Role in Synthesis
Present study	Adult retrospective single-institution cohort; analytic subset of temporary tracheostomy	*n* = 363	*n* = 61	Quantitative (primary + secondary outcomes)
Silva-Nash 2023 [30]	Adult retrospective cohort; 30-day outcomes	*n* = 317	*n* = 201	Quantitative (false passage, bleeding, infection, pneumothorax, mucus plug)
Kennedy 2021 [29]	Adult retrospective review (2015–2019)	*n* = 113	*n* = 104	Quantitative (bleeding)
Chauhan 2023 [28]	Prospective comparative study; adult elective tracheotomy	*n* = 30	*n* = 30	Quantitative (multiple complications)
İnan 2025 [31]	Adult retrospective; early (7-day) complications	*n* = 47	*n* = 117	Quantitative (tube displacement, false passage, infection, emphysema)
Çelikal 2022 [32]	Pediatric retrospective; 3 techniques	*n* = 19	VITS *n* = 24; VIMS *n* = 19	Qualitative only (pediatric, excluded from primary meta-analysis)
Marini 2023 [33]	Prospective; prolonged mechanical ventilation/critical care	*n* = 38	*n* = 41	Qualitative only (arm-specific decannulation counts not accessible)

## Data Availability

The data that support the findings of this study are available from the corresponding author upon reasonable request. The data are not publicly available owing to institutional privacy and ethical restrictions. The extracted datasets and R analysis scripts used for the meta-analysis are available from the corresponding author upon reasonable request.

## Supplementary Materials

The following supporting information can be downloaded at: https://www.mdpi.com/article/10.3390/medsci14040442/s1, Table S1: Annual accidental-decannulation events and tracheostomy volume; Table S2: Per-study outcome definitions and ascertainment; Table S3: Risk-of-bias assessment (ROBINS-I and Newcastle–Ottawa Scale); Table S4: GRADE Summary of Findings; Table S5: PRISMA 2020 checklist; Figure S1: Institutional cohort flow diagram; Figure S2: Risk-of-bias traffic-light summary (ROBINS-I).

## Appendix A. Complete Electronic Search Strategy

Three databases were searched from inception through 31 December 2025. No language restrictions were applied. The search combined controlled vocabulary and free-text terms for the procedure, the Björk flap technique, and relevant outcomes.

### Appendix A.1. PubMed/MEDLINE

#1 “Tracheostomy”[Mesh] OR “Tracheotomy”[Mesh]
#2 (tracheostom*[tiab] OR tracheotom*[tiab])
#3 (“Bjork flap”[tiab] OR “Bjoerk flap”[tiab] OR “inferior* based flap”[tiab] OR “tracheal flap”[tiab] OR “tracheal cartilage flap”[tiab] OR “inferiorly based”[tiab])
#4 (“accidental decannulation”[tiab] OR “tube displacement”[tiab] OR “false passage”[tiab] OR “false tract”[tiab] OR “tracheal stenosis”[tiab] OR complication*[tiab])
#5 (#1 OR #2) AND #3 AND #4

### Appendix A.2. EMBASE (Elsevier)

#1 ‘tracheostomy’/exp OR ‘tracheotomy’/exp
#2 (tracheostom* OR tracheotom*):ti,ab
#3 (‘bjork flap’ OR ‘inferiorly based flap’ OR ‘tracheal flap’ OR ‘tracheal cartilage flap’):ti,ab
#4 (‘accidental decannulation’ OR ‘tube displacement’ OR ‘false passage’ OR ‘tracheal stenosis’ OR complication*):ti,ab
#5 (#1 OR #2) AND #3 AND #4

### Appendix A.3. Cochrane Central Register of Controlled Trials (CENTRAL)

#1 MeSH descriptor: [Tracheostomy] explode all trees
#2 MeSH descriptor: [Tracheotomy] explode all trees
#3 (tracheostom* OR tracheotom*):ti,ab,kw
#4 (“Bjork flap” OR “tracheal flap” OR “inferiorly based flap”):ti,ab,kw
#5 (“accidental decannulation” OR “tube displacement” OR “false passage” OR complication*):ti,ab,kw
#6 (#1 OR #2 OR #3) AND #4 AND #5

Records identified: PubMed = 192; EMBASE = 112; Cochrane CENTRAL = 1 (total 305). After removal of 53 duplicates, 252 records underwent title/abstract screening. Reference lists of included studies and relevant reviews were hand-searched. See Figure 4 (main text) for the PRISMA 2020 flow diagram.
