# Supplementary material for: Inferiorly Based Björk Flap and Accidental Decannulation in Surgical Tracheostomy: A Single-Institution Cohort Study and Meta-Analysis
Source: Med Sci (Basel). 2026 Jul 27;14(4):442. doi: 10.3390/medsci14040442 (PMC13515680; doi:10.3390/medsci14040442)
Supplement: Supplementary file 1 [file medsci-14-00442-s001.zip › Supplementary_Materials.pdf]

Supplementary Materials

Inferiorly Based Björk Flap and Accidental Decannulation in Surgical Tracheostomy:
A Single-Institution Cohort Study with Meta-Analysis

Contents

Table S1. Annual accidental-decannulation events and tracheostomy volume
Table S2. Per-study outcome definitions and ascertainment
Table S3. Risk-of-bias assessment (ROBINS-I and Newcastle–Ottawa Scale)
Table S4. GRADE Summary of Findings
Figure S1. Institutional cohort flow diagram
Figure S2. Risk-of-bias traffic-light summary (ROBINS-I)

Table S1. Annual Accidental-Decannulation Events and Tracheostomy Volume

Year	Accidental-decannulation events	Total procedures	Rate (per 100)
2011	2	30	6.67
2012	1	51	1.96
2013	1	38	2.63
2014	0	60	0.00
2015	1	86	1.16
2016	0	104	0.00
2017	0	104	0.00
2018	0	130	0.00
2019	0	129	0.00
2020	0	129	0.00
2021	0	145	0.00
2022	0	133	0.00
2023	0	142	0.00
2024	0	73	0.00
2025	0	101	0.00
2026	0	30	0.00
**Pre-protocol (2011–2014)**	**4**	**179**	**2.23**
**Post-protocol (2015–2026)**	**1**	**1,306**	**0.08**

The Björk-flap safety bundle was implemented in 2015. Incidence-rate ratio (post- vs. pre-protocol) = 0.04 (95% CI 0.005–0.376). Within the per-outcome analytic subset, accidental decannulation occurred in 1/363 Björk vs. 3/61 conventional patients (OR 0.05, 95% CI 0.01–0.52; Fisher exact p = 0.010). The previously reported "per tracheostomy-years" metric has been removed in favor of events per 100 procedures.

Table S2. Per-Study Outcome Definitions and Ascertainment

Study	Outcome domain	Definition as reported	Ascertainment / timing
Silva-Nash (2023)	Primary	False passage of tracheostomy tube	Chart review; ≤30 days
İnan (2025)	Primary	Post-reinsertion tube displacement	Chart review; ≤7 days
Ajou Univ. (current)	Primary	Accidental decannulation requiring emergent intervention	Chart review; ≤14 days
Chauhan (2023)	Bleeding	Primary postoperative hemorrhage	Prospective; perioperative
Kennedy (2021)	Bleeding	Postoperative bleeding requiring intervention	Chart review; perioperative
Silva-Nash (2023)	Infection	Stomal infection / pneumonia	Chart review; ≤30 days
Chauhan (2023)	Granulation	Stomal granulation requiring intervention	Prospective; delayed
İnan (2025)	Emphysema	Subcutaneous emphysema / pneumothorax	Chart review; ≤7 days

Outcome definitions differed across studies, requiring interpretive grouping into composite domains. All three primary-domain events represent loss of secure airway access in the immature tract, the shared mechanism the Björk flap is designed to mitigate. A restricted sensitivity analysis limited to the most comparable early accidental-decannulation and false-passage definitions (excluding the İnan 2025 post-reinsertion tube-displacement dataset) is reported in the main text.

Table S3. Risk-of-Bias Assessment (ROBINS-I and Newcastle–Ottawa Scale)

Study	D1	D2	D3	D4	D5	D6	D7	Overall	NOS
Chauhan & Kumar (2023)	M	L	L	L	L	M	L	Moderate	7/9
Kennedy et al. (2021)	S	M	L	L	M	M	L	Serious	6/9
Silva-Nash et al. (2023)	S	M	L	L	M	M	L	Serious	6/9
İnan et al. (2025)	S	M	L	L	L	M	L	Serious	6/9
Ajou Univ. (current study)	S	S	L	L	M	M	M	Serious	6/9

ROBINS-I domains: D1 confounding; D2 selection of participants; D3 classification of interventions; D4 deviations from intended interventions; D5 missing data; D6 measurement of outcome; D7 selection of the reported result. L, Low; M, Moderate; S, Serious. NOS, Newcastle–Ottawa Scale (maximum 9). Overall judgments were driven predominantly by the confounding (D1) and selection (D2) domains. See Figure S2 for the graphical summary. Çelikal (2022, pediatric) and Marini (2023, decannulation counts not extractable) contributed to qualitative synthesis only and are not shown.

Table S4. GRADE Summary of Findings

Björk (inferior-based tracheal) flap versus conventional vertical-incision tracheostomy. All contributing studies were observational; certainty therefore began at "Low" per GRADE guidance and was downgraded as indicated.

Outcome domain	No. of studies (participants)	Pooled OR (95% CI); p	Certainty (GRADE)	Reasons for downgrading
Accidental decannulation / tube displacement / false passage (primary)	3 (1,168)	0.44 (0.03–5.86); $p = 0.531$	Very low	Serious risk of bias (−1); serious inconsistency, $I^2 = 77\%$ (−1); serious imprecision (−1)
Bleeding / hemorrhage	4 (638)	0.59 (0.18–1.88); $p = 0.370$	Very low	Serious imprecision, wide CI (−1); inconsistency, $I^2 = 46\%$
Infection (stomal infection / pneumonia)	3 (578)	0.26 (0.03–1.98); $p = 0.192$	Very low	Serious inconsistency, $I^2 = 77\%$ (−1); serious imprecision (−1)
Subcutaneous emphysema / pneumothorax	2 (243)	0.38 (0.07–2.10); $p = 0.268$	Very low	Serious imprecision, sparse events (−1)
Granulation / suprastomal pathology	2 (578)	0.15 (0.01–1.47); $p = 0.103$	Very low	Serious inconsistency, $I^2 = 78\%$ (−1); serious imprecision (−1)
Tube blockage / mucus plug	2 (243)	0.21 (0.01–5.22); $p = 0.343$	Very low	Serious inconsistency, $I^2 = 81\%$ (−1); serious imprecision (−1)

CI, confidence interval; OR, odds ratio. A "moderate" ROBINS-I rating was not further downgraded, to avoid double-counting the observational design; only "serious" risk of bias incurred an additional downgrade. All six domains showed directionally favorable but non-significant pooled estimates; the certainty of evidence was very low across all outcomes, and the findings should be interpreted accordingly.

Figure S1. Institutional Cohort Flow Diagram

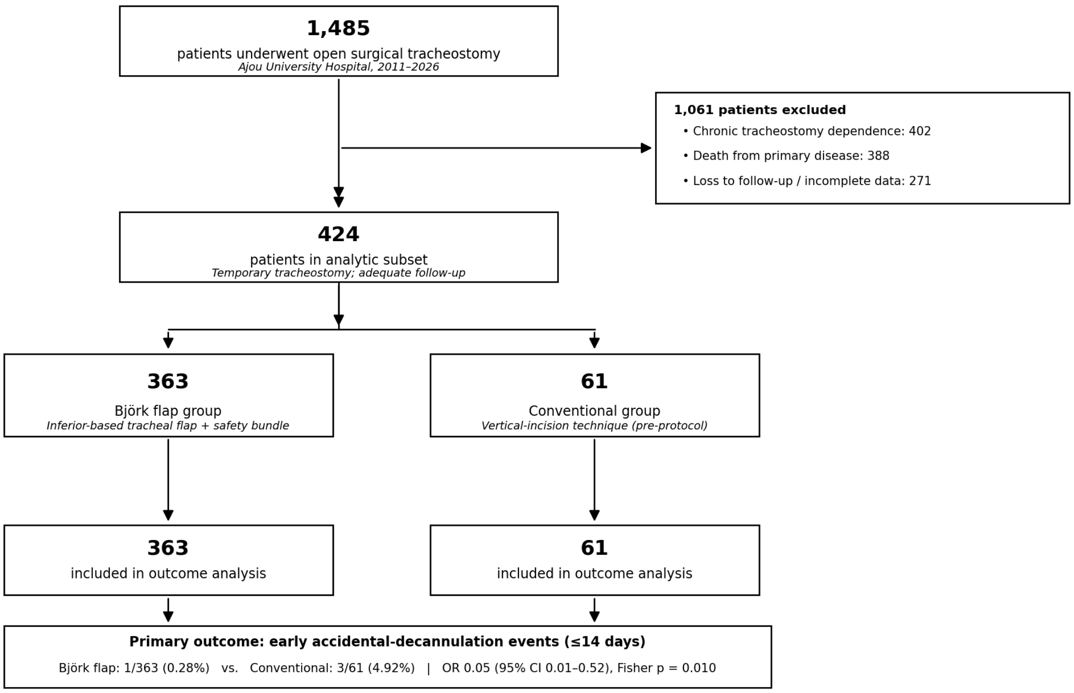

Derivation of the 424-patient analytic subset from 1,485 screened patients undergoing open surgical tracheostomy.

Figure S2. Risk-of-Bias Traffic-Light Summary (ROBINS-I)

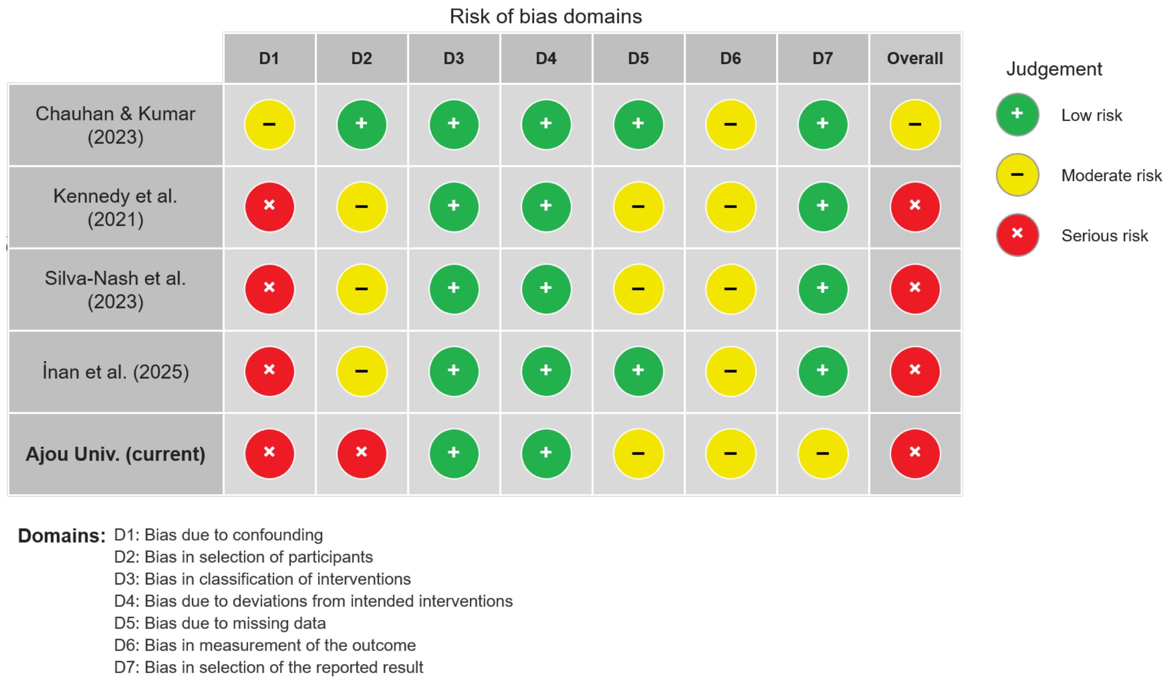

Domain-level and overall risk-of-bias judgments for the five comparative studies contributing arm-specific event data to the meta-analysis.
